# Carboxypeptidase E is a prognostic biomarker co-expressed with osteoblastic genes in osteosarcoma

**DOI:** 10.7717/peerj.15814

**Published:** 2023-08-30

**Authors:** Dafu Chen, Ben Wan, Yuning Cheng, Yuwen Luo, Xueshan Bai, Jianxun Guo, Guangping Li, Tao Jin, Jingjun Nie, Weifeng Liu, Renxian Wang

**Affiliations:** 1 Laboratory of Bone Tissue Engineering, Beijing Laboratory of Biomedical Materials, National Center for Orthopaedics, Beijing Research Institute of Traumatology and Orthopaedics, Beijing Jishuitan Hospital, Capital Medical University, Beijing, China; 2 Department of Oral and Maxillofacial Surgery, Leiden University Medical Center (LUMC), Leiden, The Netherlands; 3 Cranio-Maxillo-Facial Surgery Department, Plastic Surgery Hospital, Chinese Academy of Medical Sciences & Peking Union Medical College, Beijing, China; 4 Depatment of Orthopaedic Oncology Surgery, National Center for Orthopaedics, Beijing JiShuiTan Hospital, Capital Medical University, Beijing, China; 5 JST Sarcopenia Research Centre, National Center for Orthopaedics, Beijing Research Institute of Traumatology and Orthopaedics, Beijing Jishuitan Hospital, Capital Medical University, Beijing, China

**Keywords:** Osteosarcoma, Carboxypeptidase E, mRNA microarray, Prognostic genes, Differentially, Expressed genes, Single-cell genomics

## Abstract

Osteosarcoma (OS) is a rare primary malignant bone tumor in adolescents and children with a poor prognosis. The identification of prognostic genes lags far behind advancements in treatment. In this study, we identified differential genes using mRNA microarray analysis of five paired OS tissues. Hub genes, gene set enrichment analysis, and pathway analysis were performed to gain insight into the pathway alterations of OS. Prognostic genes were screened using the Therapeutically Applicable Research to Generate Effective Treatments (TARGET) dataset, then overlapped with the differential gene dataset. The carboxypeptidase E (CPE) gene, found to be an independent risk factor, was further validated using RT-PCR and Gene Expression Omnibus (GEO) datasets. Additionally, we explored the specific expression of CPE in OS tissues by reanalyzing single-cell genomics. Interestingly, CPE was found to be co-expressed with osteoblast lineage cell clusters that expressed RUNX2, SP7, SPP1, and IBSP marker genes in OS. These results suggest that CPE could serve as a prognostic factor in osteoblastic OS and should be further investigated as a potential therapeutic target.

## Introduction

Osteosarcoma (OS) is an aggressive pediatric bone cancer that affects the long bones near the growth plates (Gill & Gorlick, 2021). The average 5-year survival rate of localized disease is more than 60%, while the survival of patients with metastatic OS is still 10–20% (Gill & Gorlick, 2021; Zamborsky et al., 2019). The current standard OS therapy (surgery and chemotherapy) has not increased the survival rate in the many years it has been used, which may be attributed to the heterogeneous nature of the malignancy (Wan et al., 2021). An effective treatment for OS requires comprehensive molecular profiling to screen for therapeutic and predictive biomarkers.

Previous studies have investigated many prognostic factors (mRNA, noncoding RNAs, cytokines, serum proteins) for OS (Zamborsky et al., 2019). Zhang, Liu & Kong (2021) reported mRNAs and lncRNA candidate prognostic biomarkers (LINC00957, METL1, CA9, B3GALT4, ALDH1A1, LAMB3 and ITGB4) for recurrence in patients with OS. Bhatti et al. (2022) found that serum alkaline phosphatase served as a biomarker with high specificity in OS patients. Moreover, Lewis et al. (2017) revealed that the expression of IL-11R^3^ correlated with poor prognosis in OS. However, due to the small sample size and lack of properly controlled randomized clinical studies, few markers are reliably used to monitor OS progression or predict prognosis. Shi et al. (2020) downloaded an OS mRNA dataset from the Gene Expression Omnibus (GEO), and identified three-gene survival risk signatures (MYC, CPE, and LY86) by bioinformatics methodology. The above feature gene analyses of tumors are currently based on microarray and next-generation sequencing data of bulk tumor samples.

Conventional bulk RNA profiling captures the average expression states of all cells within a sample, but it remains difficult to characterize heterogeneous cells with distinct genetic and phenotypic properties (Lawson et al., 2018). Single-cell RNA sequencing (RNA-SEQ) has been widely used to provide genome-wide single-cell granularity for mRNA expression analysis, and has been explored in OS (Lawson et al., 2018). Feleke et al. (2022) identified four differentially expressed survival-related genes (MUC1, COL13A1, JAG2, and KAZALD1) using the Therapeutically Applicable Research to Generate Effective Treatments dataset (Feleke et al., 2022; Liu et al., 2021). Furthermore, Qin et al. (2022) reported the potential prognostic value of ATG16L1 in OS using conventional bulk RNA sequencing and scRNA-Seq. Therefore, integrating bulk RNASeq and scRNA-Seq analyses is a prospective method for characterizing the composition of the tumor microenvironment and screening new biomarkers.

The study of Shi et al. (2020) suggested carboxypeptidase E may be a promising prognostic biomarker for OS. In humans, carboxypeptidase E (CPE), a member of the M14 family of metallic carboxypeptidases, encodes carboxypeptidases that cleaver C-terminal amino acid residues (Hareendran et al., 2022). CPE is involved in the biosynthesis of peptide hormones and neurotransmitters, including insulin. Previous researches have found that CPE acts a role in tumorigenesis and cancer progression (Hareendran et al., 2022). Höring et al. (2012) reported that the expression of CPE was reduced in glioblastoma and was hypothesized to be a tumor suppressor gene. In contrast, CPE is elevated in colorectal cancer and plays a vital role in cell cycle regulation (Liang et al., 2013). CPE gene expression has been shown to be upregulated in OS samples when compared to healthy controls, indicating the role that CPE plays in the development of OS (Yang et al., 2014). Indeed, Fan et al. (2016) suggested that CPE is correlated with cell growth, tumorigenicity, migration, and invasiveness in OS cells. However, the landscape of abnormally-expressed CPE in OS subpopulations remains unclear.

In this study, we explored mRNA expression profiles *via* microarray data from five paired tissue samples to reveal molecular aberrations. Based on differentially expressed genes and a public database, we determined that CPE was enriched only in the osteoblastic cell population of OS tissue samples.

## Materials and Methods

### Patient samples

This study was conducted on osteosarcoma tissues and adjacent normal tissues collected from the Department of Orthopaedic Oncology Surgery of the Beijing JiShuiTan Hospital. All of the patients had received four cycles of standard preoperative adjuvant chemotherapy (high-dose methotrexate, ifosfamide, cisplatin, and doxorubicin, MAP + IFO) prior to tissue collection (Suehara et al., 2019). The tissue samples were stored in liquid nitrogen following surgery (Table 1). Sample was collected with written informed consent from patient’s family members, according to the ethics committee of Beijing Jishuitan Hospital approved protocols (Grant No. K2022-117-00).

**Table 1:** Patient characteristics.

Patient	Metastasis	Age	Sex	Location	Outcome	Enneking stage
1	Lung, Soft tissue	27	F	Dorsal metastases	DOD	III
2	Lung	13	F	Proximal of tibia	DOD	III
3	Lung	15	M	Proximal of tibia	SWT	III
4	N	12	M	Distal of femur	NED	IIB
5	N	19	M	Distal of femur	NED	IIB
6	N	14	M	Proximal of tibia	NED	IIB
7	N	27	F	Distal of femur	NED	IIB
8	N	56	F	Distal of femur	NED	IIB
9	N	13	F	Proximal of tibia	NED	IIB
10	N	65	F	Distal of femur	SWT	IIB
11	N	12	F	Proximal of femur	NED	IIB
12	N	13	M	Distal of femur	NED	IIB
13	Lung	18	M	Proximal of femur	DOD	III
14	Lung	69	F	Proximal of tibia	DOD	IIB
15	Lung	20	M	Proximal of tibia	SWT	IIB
16	N	15	F	Proximal of femur	NED	IIB
17	N	22	M	Distal of femur	NED	IIB
18	Lung, Brain	13	M	Distal of femur	DOD	III
19	Humerus	13	M	Femur	SWT	III
20	Breast	15	F	Femur	DOD	III

**Notes:**

Twenty paired OS samples (Table 1) were subjected to quantitative reverse transcription-PCR (qRT-PCR) to determine the expression of CPE (NM_001873.4), TRSP1 (NM_001330599.2), PFKFB3 (XM_047425347.1), and FADS1 (XM_047426935.1).

NED, No Evidence of Disease; SWT, Survival With Tumor; DOD, Dead of Disease.

### Filtering of DEG from microarray

Agilent Human lncRNA Microarray 2019 (4*180k, Design ID: 086188) was used. Data analysis of 12 samples was completed by OE Biotechnology Co., Ltd., (Shanghai, China). Quantification of total RNA was carried out using a spectrophotometer, NanoDrop ND-2000 (Thermo Fisher Scientific, Waltham, MA, USA), while the integrity analysis of RNA samples was performed using an RNA analyzer, Agilent Bioanalyzer 2100 (Agilent Technologies, Santa Clara, CA, USA). The subsequent steps, including sample labeling, microarray hybridization, and washing, were executed following established protocols recommended by the manufacturer. In brief, the total RNA was subjected to reverse transcription to synthesize double-stranded cDNA, which was then converted into complementary RNA (cRNA) and labeled using Cyanine-3-CTP. The labeled cRNAs were subsequently hybridized onto a microarray, and following a thorough washing step, the microarrays were scanned using the Agilent Scanner G2505C (Agilent Technologies, Santa Clara, CA, USA).

The whole-gene expression datasets were subjected to principal component analysis (PCA) for dimensionality reduction and quality control. One subject was excluded due to poor clustering. We conducted differential gene analysis using the R software “limma” package (https://bioconductor.org/packages/limma/) according to the filter criteria (log2|fold change| > 2, FDR < 0.05). Subsequently, the gene expression profile and survival (survival state and survival time) of patients with OS were analyzed by Cox regression, and prognostic related genes were detected in the TARGET database. Screening high risk factors related to survival based on *P* < 0.05.

### Protein-protein interaction network and hub gene

STRING (https://string-db.org/) was employed to predict interactions between functional proteins and to establish a PPI network. The network was conducted with Cytoscape software (version 3.9.1). The PPIs between the putative protein biomarkers were analyzed using the STRING web service database (https://string-db.org/). Hub gene analysis was conducted after considering the high degree of connectivity in the PPI networks analyzed by the Maximal Clique Centrality (MCC) plugin of Cytoscape (Chin et al., 2014). Here, the top 10 MCC values were considered hub genes and were presented in the form of a PPI network.

### Evaluation of the expression level of CPE

Two published OS datasets (GSE126209, GSE33382) were obtained from the GEO database for the expression-level evaluation of CPE. We selected 11 paired samples from the GSE126209 series and 68 OS samples, including three pathological types of OS (“Fibroblastic” 8, “Osteoblastic” 51, “Chondroblastic” 9), from GSE33382 series.

### Total RNA extraction and qRT−PCR

Twenty paired OS samples (Table 1) were subjected to quantitative reverse transcription-PCR (qRT-PCR) to determine the expression of CPE (NM_001873.4), TRSP1 (NM_001330599.2), PFKFB3 (XM_047425347.1), and FADS1 (XM_047426935.1). In accordance with the manufacturer’s instructions, TRIzol reagent (Invitrogen, Waltham, MA, USA) was used to extract total RNA (Invitrogen, Waltham, MA, USA). RT-PCR analysis was performed using the LightCycler® 480 Probes Master Mix (LightCycler® 480II; Roche, Basel, Switzerland). RT-qPCR was performed using a 2720 thermal cycler (Applied Biosystems, Waltham, MA, USA). The relative mRNA levels were normalized against the housekeeping gene GAPDH (NM_001357943.2). PCR primer sequences are summarized as follows: GAPDH(F)5′-TGAAGGTCGGAGTCAACGG-3′,(R): 5′-TCCTGGAAGATGGTGATGGG-3′; TRSP1(F): 5′-AGCCCCAGTAAGGGAGGAAA-3′,(R): 5′-GGGTGCAGGCCATATCTTGAG-3′; PFKFB3(F):5′-GATGCCCTTCAGGAAAGCCT-3′,(R): 5′-TTGAACACTTTTGTGGGGACGC-3′;FADS1(F): 5′-GTCTTCTTCCTGCTGTACCTG-3′,(R): 5′-GGTTCCACTTTGAGGTGCTGA-3′; CPE(F): 5′-CATCTCCTTCGAGTACCACCG-3′,(R): 5′-CCGTGTAAATCCTGCTGATGG-3′.

### Gene function analysis and survival analysis

The top 50 hub genes for Gene Ontology (GO) and Kyoto Encyclopedia of Genes and Genomes (KEGG) analysis was built using R. *P* < 0.05 was the cutoff criterion, and RUNX2, IBSP, SPP1, SP7 were selected as osteogenic marker genes (Liu et al., 2020). Pearson correlation coefficients were used to indicate the correlation between the marker genes and CPE. The pROC (v 1.17.0.1) analysis was used to compare the predictive accuracy of CPE. The TARGET RNA sequencing data were used to divide patients into low- and high-expression groups according to the median relative gene expression level (50%). Kaplan–Meier survival analysis were conducted with the TARGET cohort in the Xiantao platform.

### Analysis of scRNA-Seq transcriptome datasets

The scRNA-Seq transcriptome datasets for GSE162454 were from the NCBI website (https://www.ncbi.nlm.nih.gov/geo). The data quality control process was analyzed using the Seurat package (version 4.1.1). The Merge function was used to merge six OS cases. Quality control was performed using the following criteria: the feature gene number range from 300 to 4,500, and gene counts <15,000 and those with a mitochondrial gene number of <10%. Subsequently the Harmony package (version 1.0) was used to eliminate the batch effect of the cellular data. In addition, the FindClusters function of the Seurat package was used for cell clustering analysis (resolution = 0.091) and visualization by performing a uniform manifold approximation and projection (UMAP) dimension reduction analysis. The cell populations were annotated based on their respective gene expression levels in a known set of genes reported by Liu et al. (2021) (Table S1). The average gene expression was visualized using the VlnPlot and FeaturePlot functions of the Seurat packageS.

### Statistical analysis

All analysis methods and R packages were implemented with R version 4.1.3 (R Core Team, 2022). Wilcoxon matched-pairs signed-rank test was used for the comparison between tumor and the adjacent tissues, while Student’s *t*-test was used to compare the two groups. *P*-values < 0.05 were considered statistically significant.

## Results

### Identification of DEGs and hub genes in OS samples

**Figure 1:**
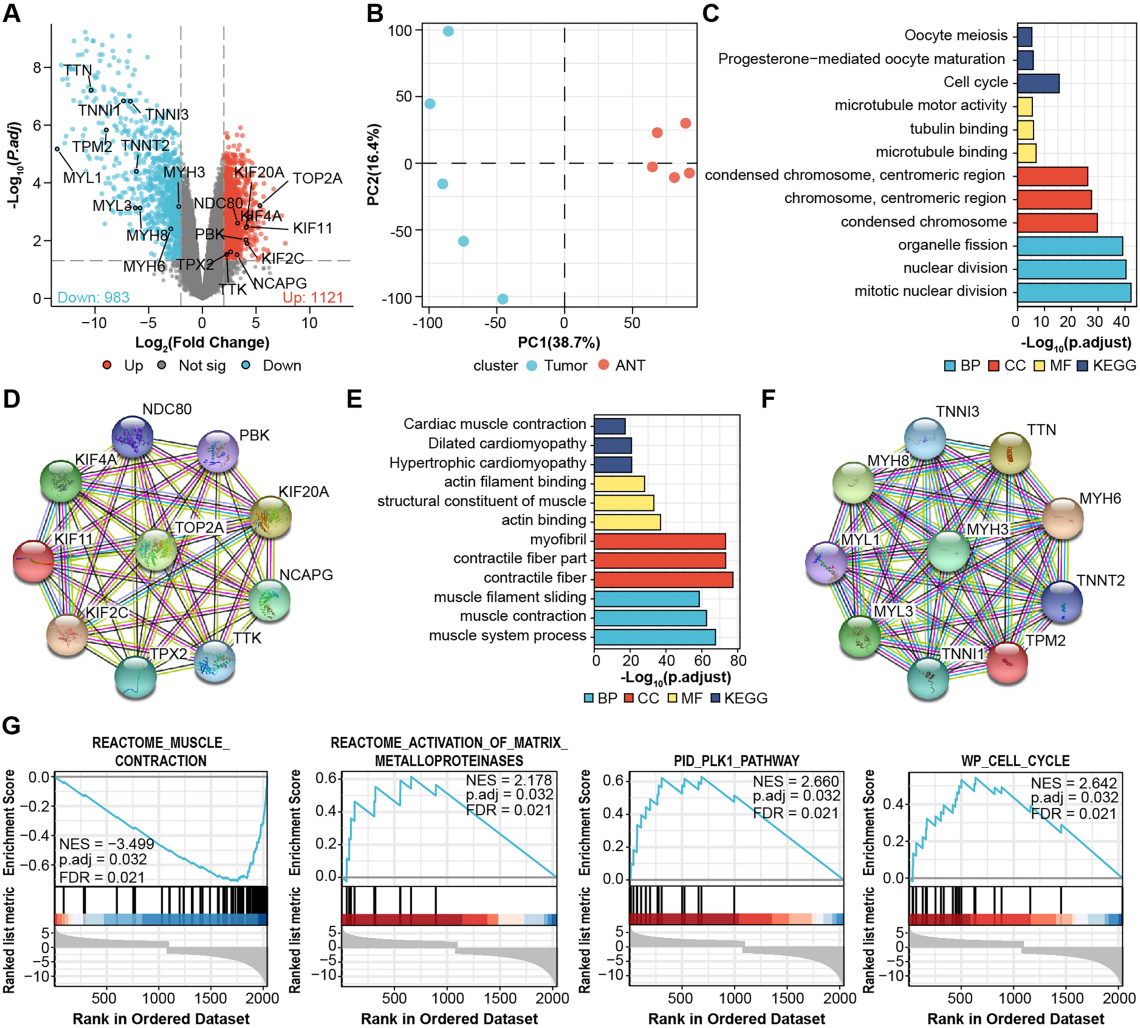
Identification of DEGs from microarray. (A) Volcano plots for DEGs and labeled with top 10 hub genes. Cut-off value: log2|fold change| > 2, FDR < 0.05. (B) PCA, principal component analysis. (C) GO, KEGG results of top 50 upregulated hub genes. (D) PPI network of top 10 upregulated hub gene. (E) GO, KEGG results of top 50 downregulated hub genes. (F) PPI network of top 10 downregulated hub gene. Annotations for Go results are grouped by biological process, cellular component, and molecular function. (G) GSEA enrichment plots of DEGs.

We performed microarray analysis to screen differential genes between OS samples and adjacent tissues. The volcano plot shows that 1,121 genes were upregulated and 983 genes were downregulated (Fig. 1A, File S1). PCA showed sample clustering by DEGs (Fig. 1B), indicating that the tumor tissue and adjacent normal tissue had different expression patterns. We next analyzed the DEGs using GO and enrichment in KEGG pathways using the top 50 hub genes (Figs. 1C and 1E). The most significantly enriched terms in upregulated genes were “Cell cycle” in KEGG (Fig. 1C), “microtubule binding” in GO molecular function, “condensed chromosome” in GO cellular component, and “mitotic nuclear division” in GO biological process. In the downregulated genes, the annotation items were related to skeletal muscle physiological activity. The top 10 upregulated hub genes (KIF11, KIF20A, KIF2C, KIF4A, NCAPG, NDC80, PBK, TOP2A, TPX2, TTK) are presented in the PPI network (Fig. 1D). The top 10 downregulated hub genes were TTN, MYH6, TNNI, MYH8, MYH3, MYL1, TNNT2, TNNI3, TPM2, and MYL3 (Fig. 1F).

We then performed Gene-Set Enrichment Analysis (GSEA) with all genes (Fig. 1G, File S2). These hallmark gene sets were significantly enriched in “reactome_muscle_contraction” (NES = 3.499; p.adjust = 0.032; FDR = 0.021), “reactome_activation_of_ matrix_metalloprotein” (NES = 2.178; p.adjust = 0.032; FDR = 0.021), “PID_PLK1_pathway” (NES = 2.66; p.adjust = 0.032; FDR = 0.021), and “WP_cell_cycle” (NES = 2.642; p.adjust = 0.032; FDR = 0.021), suggesting the progression of osteosarcoma is related to these processes.

### Screening for prognostic genes

**Figure 2:**
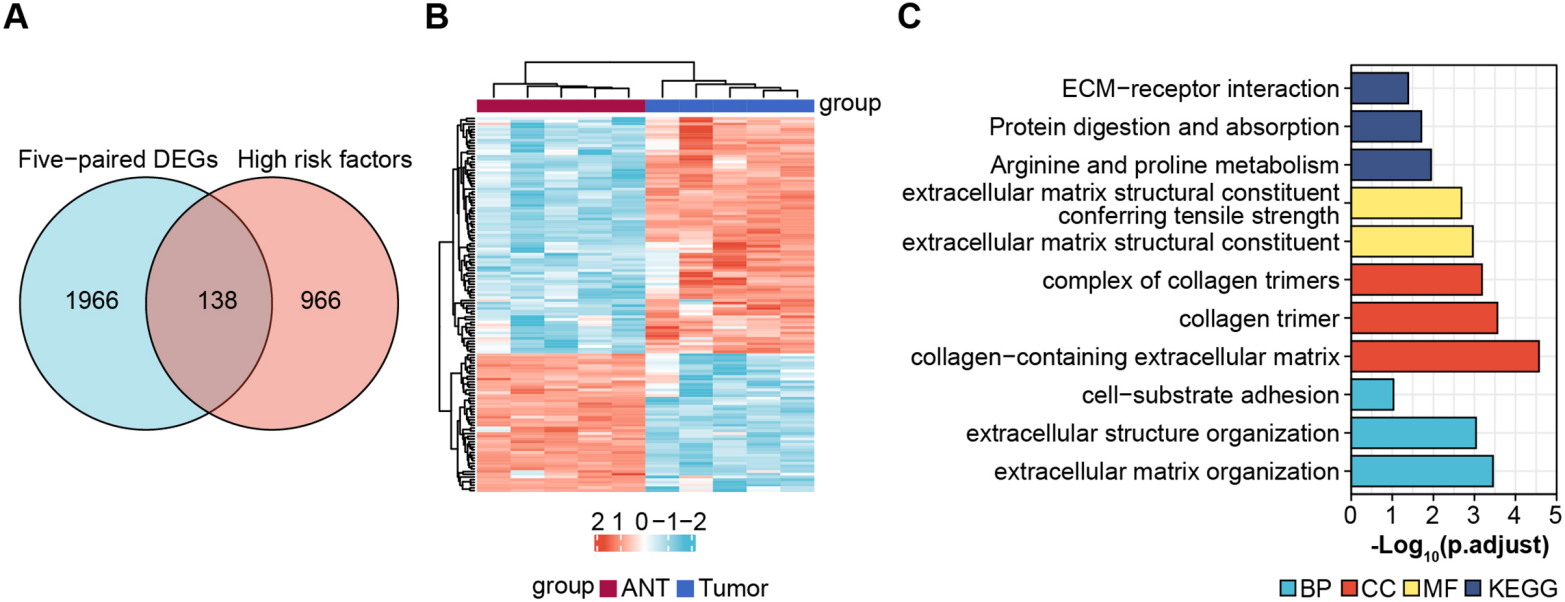
Screening prognostic genes. (A) Veen plot for screening prognostic genes by overlapping DEGs and high risk factors, cut-off value: *P*-value < 0.05. (B) Heat map of prognostic genes. (C) GO and KEGG for functional annotation of the 138 aberrant prognostic genes.

**Figure 3:**
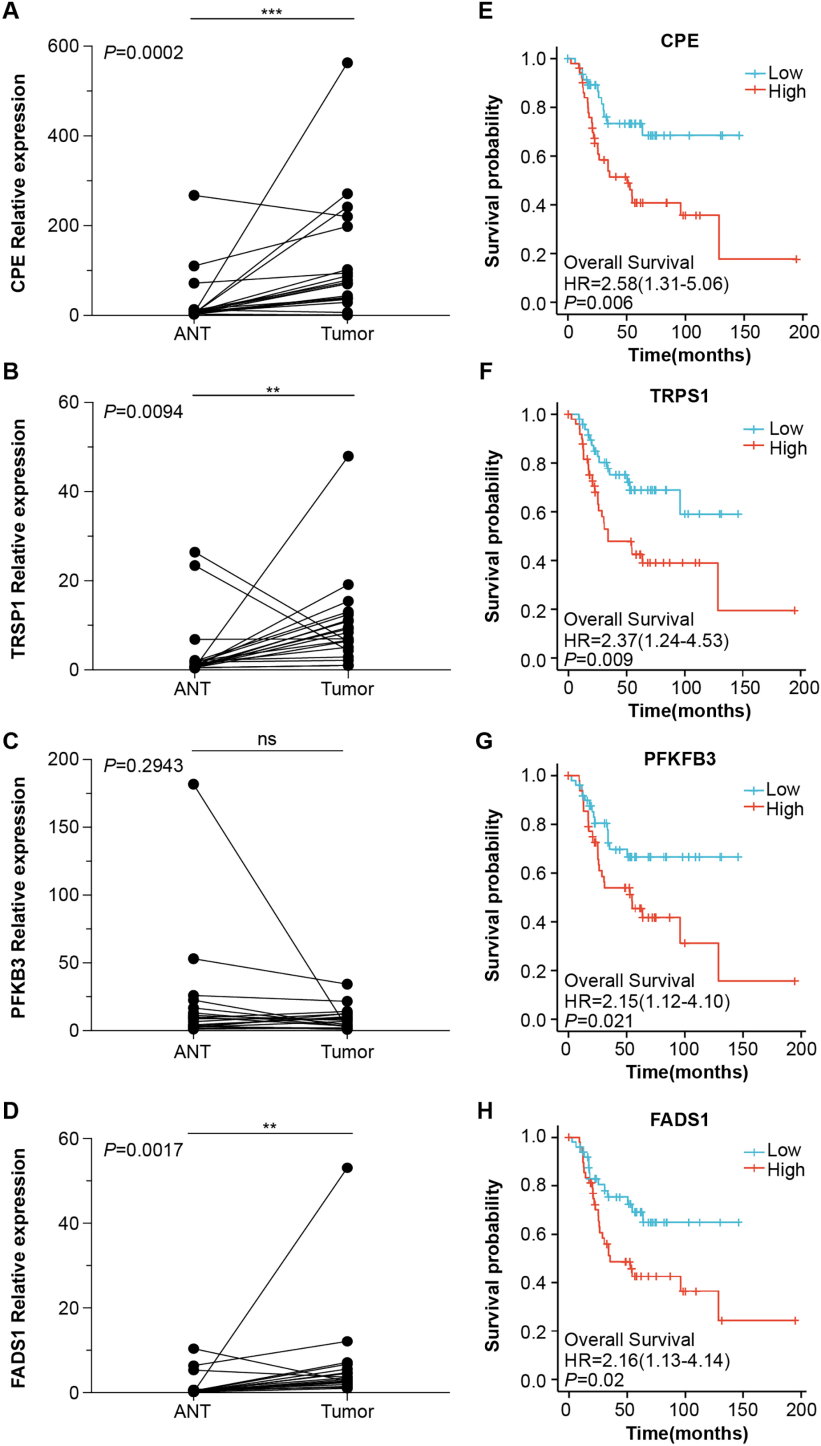
Four abnormal gene expression were associated with overall survival rate. The expression level of (A) CPE, (B) TRPS1, (C) PFKFB3, (D)FADS1, was determined by RT-qPCR in 20 paired tumor and adjacent normal tissues from OS patients. Kaplan–Meier survival analysis of (E) CPE, (F) TRPS1, (G) PFKFB3, (H)FADS1. **P-value < 0.01 and ***P-value < 0.001.

Next, we obtained 1,104 high-risk factors from the TARGET database by Cox regression analysis (Fig. 2A, File S3). We overlapped the DEGs with the high-risk factors and presented the relative expression levels of the 138 dysregulated prognostic genes in the heatmap (Fig. 2B). These genes were highly enriched for extracellular matrix-related items (Fig. 2C). We validated four abnormal genes in the tumor tissue from the prognostic genes lists using qRT-PCR analysis (Figs. 3A–3D). The results of CPE, TRSP1, and FADS1 were in accordance with the sequencing results (logFC: 2.6767, 1.9593, 2.5813 respectively, *P* < 0.05). However, the PFKFB3 was inconsistent with high-throughput data which was downregulated in tumor tissue (logFC: −2.369, *P* < 0.05). Moreover, OS patients with higher expression of CPE, TRSP1, PFKFB3, and FADS1 had worse prognoses than those with lower expression in the TARGET database (Figs. 3E–3H). These results indicated that the CPE, TRSP1, and FADS1 may be associated with the prognosis of osteosarcoma patients and worth further research.

### CPE was highly expressed in tumor tissues

**Figure 4:**
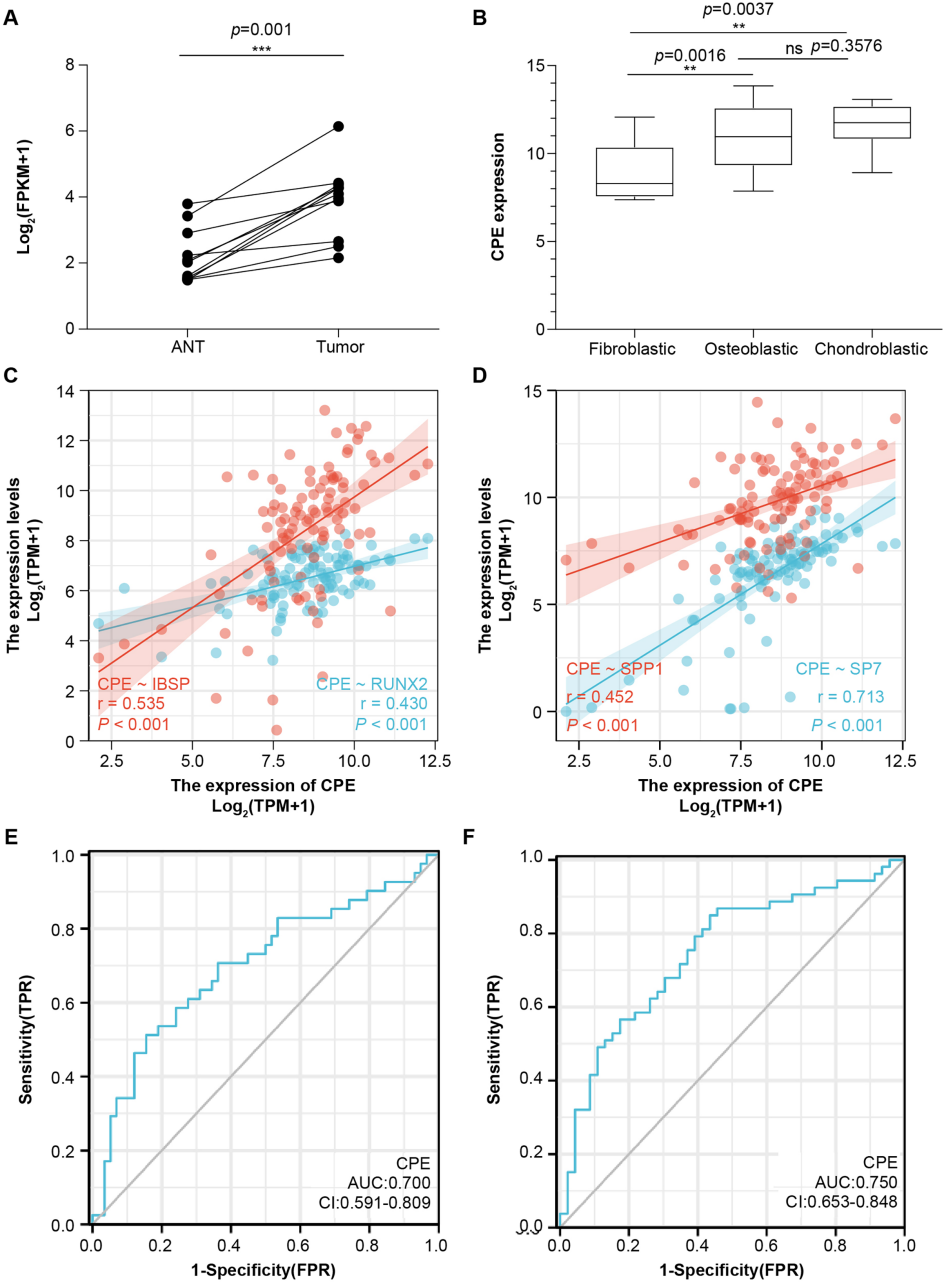
The gene signature of CPE in two GEO testing datasets and TARGET database. Relative mRNA expression level of CPE based on the 11 paired OS samples from GSE126209 (A) and 68 OS samples from GSE33382 (B). Two-gene correlation analysis results between CPE and osteogenic gene (C and D). The AUC values of the CPE gene for overall survival. (E) and relapse-free survival (F). **P-value < 0.01 and ***P-value < 0.001.

To further validate the microarray data, we used the public database to detect CPE mRNA expression (Figs. 4A and 4B). CPE was highly expressed in tumor samples, which was consistent with the results of the microarray (logFC = 2.6767, adj.P.Val = 0.0029). GSE126209 included the 11 paired mRNA profile in OS and adjacent normal tissues. The results showed a significantly higher expression in OS tissues than normal samples (*P* = 0.001, Fig. 4A). We also analyzed the relationship between CPE expression clinicopathological features in 68 OS patients from GSE33382. Both osteoblastic and chondroblastic OS had higher expression compared to fibroblastic pathological types (*P* < 0.05, Fig. 4B). We also found a coexpression pattern between the CPE and osteoblastic markers in the TARGET database (Figs. 4C and 4D). The correlation coefficients (*P* < 0.001) for RUNX2, IBSP, SPP1, and SP7 were 0.43, 0.535, 0.452, and 0.713, respectively. A correlation coefficient of SP7 (r > 0.7) was considered to indicate high correlation.

### CPE was an independent prognostic factor for OS

Next, univariate and multivariate Cox regression analyses were performed for overall survival and relapse-free survival to identify independent prognostic factors (Tables S2 and S3). The results indicated that CPE was independent of other clinical factors in relapse-free survival (*P* = 0.028), while there was no statistical difference in overall survival (*P* = 0.071), indicating that CPE was correlated with the relapse-free survival of the patients with OS. The AUC value of overall survival was 0.700 (CI [0.591–0.809], Fig. 4E), and the AUC value of relapse-free survival was 0.75 (CI [0.653–0.848], Fig. 4F). The dysregulated genes of CPE had moderate predictive power.

### CPE was strongly enriched in osteoblastic OS cells

**Figure 5:**
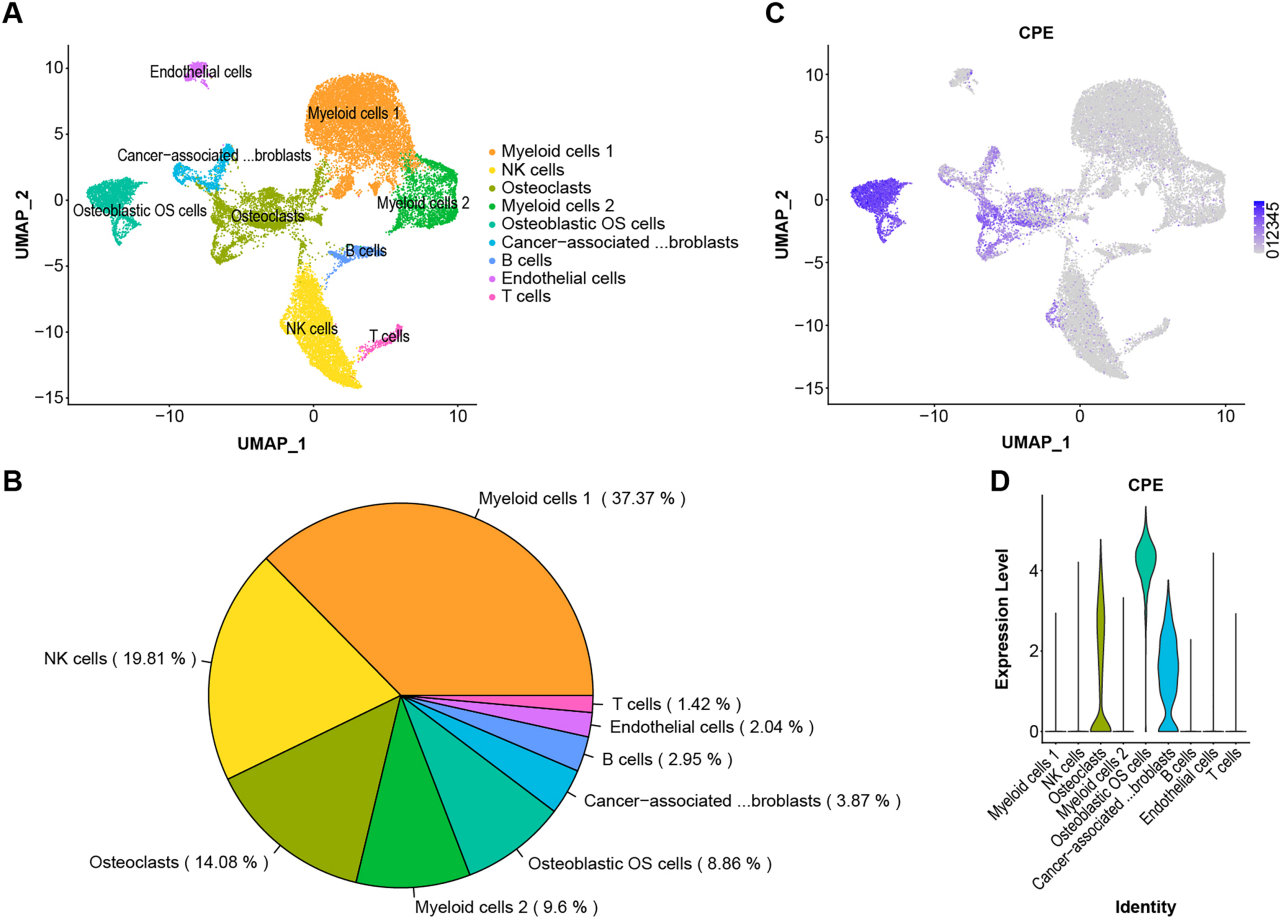
Single-cell RNA-seq identification of CPE. (A) The UMAP algorithm for dimensionality reduction in six OS samples. (B) Proportion of cell types. (C and D) The expression level of CPE in different cell populations.

The scRNA-Seq transcriptome datasets were collected from NCBI for six treatment-naive OS cases to investigate the effects of CPE genes. After normalization and PCA dimensionality reduction, the dimensionality reduction data were displayed in 2D space using Uniform Manifold Approximation and Projection (UMAP) analysis, and the cells were then overlapped into nine clusters (Figs. 5A and 5B). Subsequently, we checked the transcription levels of CPE between the different OS tumor cell types. The average expression values of CPE in all cell types were shown in Figs. 5C and 5D. Our results suggested that CPE was highly expressed in osteoblasts, osteoclast OS cells, and carcinoma-associated fibroblast clusters but expressed at low levels in other cell types.

## Discussion

OS is a highly malignant bone tumor with high cellular heterogeneity and transcriptional complexity. Many articles have characterized the possible molecular pathogenesis of OS using bioinformatics analysis of data collected from public datasets (Zamborsky et al., 2019; Wan et al., 2021; Zhang et al., 2021). By analyzing OS datasets, these researchers gained insights into the molecular functions of specific gene expression modules in the disease. In addition, single-cell genomics have provided promising new tools for exploring the tumor microenvironment. Both Zhou et al. (2020) and Liu et al. (2021) reported on the single-sell transcriptomics landscape in OS. Additionally, the survival-related genes and a highly invasive cell population have been identified using previous data (Feleke et al., 2022; Qin et al., 2022; Guo et al., 2022). One of the key findings in this work was that CPE was upregulated in the OS samples. Additionally, by screening the DEGs from the microarray and validating the results *via* RT–qPCR and the GEO dataset, we found significantly higher expression of CPE in osteoblastic OS tissues (Figs. 3A, 4A and 4B). Meanwhile, high expression of CPE predicted a worse prognosis of OS (Fig. 3E), and osteogenic genes, specifically SP7, were highly correlated with the expression of CPE (Fig. 4D). We also explored the function of the CPE gene at the single-cell level (Fig. 5).

Many articles have predicted the prognostic value of CPE in OS. Shi et al. (2020) selected metastasis associated genes using a bioinformatics approach based on TARGET datasets and four microarrays from the GEO. The results showed that high-level expression of CPE was a risk factor (hazard ratio (HR): 2.313, *P* = 0.001572) and could predict the prognosis of patients with OS. Although this result was in agreement with the results of this study, we also found that CPE was tightly correlated with relapse-free survival (HR: 2.343, *P* = 0.028). Moreover, we only included CPE to perform multivariate Cox regression analysis and found that CPE was not sufficiently powered to assess significance at 95% confidence in overall survival. Simultaneously, we also investigated whether CPE could serve as a predictive indicator for overall survival or relapse-free survival (Figs. 4E and 4F). Our results showed no significant difference in the expression level of PFKFB3 between cancer and paracancerous tissues, although Kaplan–Meier analysis suggested that low PFKFB3 expression was indicative of good prognosis (Fig. 3G). Moreover, TRSP1 and FADS1 were highly expressed in tumor samples and correlated with prognosis features (Figs. 3B and 3D). These results suggest that TRSP1 and FADS1 play roles in cancer progression. Li et al. (2015) reported that overexpression of TRSP1 contributes to poor prognosis in human OS, as detected by immunohistochemistry. Furthermore, Lian et al. (2019) used 33 paired OS samples and the TARGET database to confirm that high expression of FADS1 contributed to poor prognosis. These results were qualitatively consistent with those of the current study. Recently, several highly malignant liver, prostate, pancreatic, and glioblastoma cells cells were found to release the WT-CPE protein and mRNA in exosomes (Hareendran et al., 2022; Hareendran et al., 2022). Additionally, there was a correlation between increased CPE mRNA levels in exosomes from highly malignant and moderately malignant cancer cells. We found that CPE may be detected as a potential diagnostic/prognostic biomarker using liquid biopsy. CPE acts as an oncogene in OS. Zhang et al. (2021) performed qRT–PCR analyses and demonstrated that CPE is overexpressed in OS cell lines (143B, U2OS, and MNNG/HOS) compared to an osteoblast cell line (hFOB 1.19). Moreover, silencing of CPE has been shown to inhibit xenograft tumor growth *in vivo* by inducing cell cycle arrest (Fan et al., 2016). Overexpression of CPE-ΔN (an N-terminally truncated splice variant of CPE) in OS cells enhances cell migration, invasiveness, and promotes epithelial–mesenchymal transition (Fan et al., 2019). However, CPE is abnormally expressed in many cancers, including neuroendocrine carcinoma, cervical cancer, ovarian cancer, pancreatic cancer, hepatocellular carcinoma, glioblastoma, and colorectal cancer (Liang et al., 2013; Armento et al., 2017; Huang et al., 2016; Liu et al., 2014; Shen et al., 2016; Cawley et al., 2012; Hareendran et al., 2019). CPE is a prohormone processing enzyme that is highly secreted from (neuro) endocrine tumors, suggesting that secreted CPE may promote tumor growth (Hareendran et al., 2022). Hareendran et al. (2022) reported that the mRNA levels of CPE from serum exosomes were highly elevated in malignant cancer cells, and provided evidence that exosomal CPE promoted the transmission of tumorigenesis. Murthy et al. (2013) found that exogenous CPE could protect MHCC97H cells from metabolic stress and reduce the migration and invasion of fibrosarcoma (HT1080) cells. CPE can be secreted by many types of cancer cells and may activate oncogenic signal transduction pathways following receptor binding (Hareendran et al., 2022). Cell survival genes (*e.g*., TNF, IL8), anti-apoptotic genes (*e.g*., Bcl2), and cell cycle regulator genes (*e.g*., Cyclin D1) are upregulated because of these signaling pathways (Hareendran et al., 2022). The detailed mechanisms underlying CPE secretion still need to be elaborated in OS.

Generally, OS cases are classified as osteoblastic (50%), chondroblastic (25%), or fibroblastic (25%) (Gill & Gorlick, 2021). Even though OS has a very distinctive radiographic appearance, it is difficult to differentiate from other malignant bone tumors based on pathological features alone. According to the results, CPE can be expressed at high levels in osteoblast osteosarcoma cells and at low levels in osteoclastic and carcinoma-associated fibroblast populations (Figs. 5C and 5D). CPE and SP7 were found to be highly correlated with mRNA expression in OS (Fig. 4D), suggesting that CPE plays a key regulatory role in osteogenesis and the tumorigenicity of osteoblastic OS. Previous *in vitro* studies revealed that CPE plays an important role in RANKL-dependent osteoclast differentiation (Kim et al., 2014). Recent single-cell RNA sequencing data of human osteoblasts have suggested that CPE is enriched in mature osteoblasts, and high expression of CPE can be a feature gene marker for osteoblastic cell clusters (Gong et al., 2021). Furthermore, Cawley et al. (2010) explored low bone density in CPE knockout (KO) mice, an obese animal model. Thus, these data prove that CPE may serve as a predictive biomarker for osteoblastic OS diagnosis.

Utilizing data mining, the current study contributes to understanding the relationship between CPE expression and osteoblastic OS. This study supports previous research suggesting that CPE is a prognostic biomarker in patients with OS and plays an important role in tumorigenesis. However, this study has several limitations that warrant discussion. First, the results were based on microarray datasets and qRT–PCR results. To validate the expression and subcellular localization of CPE, more experiments need to be conducted on histopathological slides. Second, as the total number of patients with OS in all datasets was relatively small, it is necessary to conduct further clinical studies to assess the prognostic value of CPE. Third, the molecular function of CPE is unknown in osteoblastic OS, and requires more direct evidence from *in vitro* and *in vivo* experiments.

## Conclusions

To summarize, we identified a set of prognosis-related genes and determined that dysregulated CPE is a potential prognostic biomarker that is co-expressed with osteoblastic genes. Our work sheds new light on the use of CPE as a potential novel diagnostic biomarker and therapeutic target for OS.

## Supplemental Information

10.7717/peerj.15814/supp-1 Supplemental Information 1 List of differential genes detected by microarray.

10.7717/peerj.15814/supp-2 Supplemental Information 2 List of high risk factors screening based microarray.

10.7717/peerj.15814/supp-3 Supplemental Information 3 Gene List for Gene Set Enrichment Analysis(GSEA) based microarray.

10.7717/peerj.15814/supp-4 Supplemental Information 4 Annotation for major cell cluster based on marker genes.

10.7717/peerj.15814/supp-5 Supplemental Information 5 Univariate analysis and Multivariate analysis of overall survival.

10.7717/peerj.15814/supp-6 Supplemental Information 6 Univariate analysis and Multivariate analysis of relapse-free survival.

10.7717/peerj.15814/supp-7 Supplemental Information 7 Miame Checklist.

## Additional Information and Declarations

### Microarray Data Deposition

The following information was supplied regarding the deposition of microarray data: Agilent Human lncRNA Microarray 2019.

### Data Availability

The following information was supplied regarding data availability: The data is available at GEO: GSE126209, GSE33382, GSE225588.
