# Supplementary material for: Carboxypeptidase E is a prognostic biomarker co-expressed with osteoblastic genes in osteosarcoma
Source: PeerJ. 2023 Aug 30;11:e15814. doi: 10.7717/peerj.15814 (PMC10474831; doi:10.7717/peerj.15814)
Supplement: Supplemental Information 4 [file peerj-11-15814-s004.docx]

Table S1 Annotation for major cell cluster based on marker genes

| **Major cell cluster** | **Marker genes** |
| --- | --- |
| Myeloid cells | LYZ, CD68 |
| osteoblastic OS cells | ALPL, RUNX2, IBSP |
| Osteoclasts | ACP5, CTSK |
| Cancer-associated ﬁbroblasts | COL1A1, FAP, VIM |
| NK cells | NKG7, KLRD1, KLRB1 |
| Endothelial cells | EGFL7, PLVAP |
| B cells | MS4A1, CD79A, CD19 |
| T cells | CD3D, CD3E, CD79A |
