# Supplementary material for: Carboxypeptidase E is a prognostic biomarker co-expressed with osteoblastic genes in osteosarcoma
Source: PeerJ. 2023 Aug 30;11:e15814. doi: 10.7717/peerj.15814 (PMC10474831; doi:10.7717/peerj.15814)
Supplement: Supplemental Information 5 [file peerj-11-15814-s005.docx]

Table S2 Univariate analysis and Multivariate analysis of overall survival

| Characteristics | Total(N) | Univariate analysis | |  | Multivariate analysis | |
| --- | --- | --- | --- | --- | --- | --- |
|  |  | Hazard ratio (95% CI) | P value |  | Hazard ratio (95% CI) | P value |
| Metastasis | 99 |  |  |  |  |  |
| No | 75 | Reference |  |  |  |  |
| Yes | 24 | 3.679 (1.964-6.892) | **<0.001** |  | 3.320 (1.438-7.664) | **0.005** |
| Tumor region | 63 |  |  |  |  |  |
| Distal | 36 | Reference |  |  |  |  |
| Other&Proximal&Proximal & Distal | 27 | 0.473 (0.198-1.127) | 0.091 |  | 0.624 (0.257-1.516) | 0.298 |
| Age | 99 |  |  |  |  |  |
| <18 | 76 | Reference |  |  |  |  |
| >=18 | 23 | 0.732 (0.325-1.653) | 0.454 |  |  |  |
| CPE | 99 |  |  |  |  |  |
| Low | 48 | Reference |  |  |  |  |
| High | 51 | 2.580 (1.315-5.064) | **0.006** |  | 2.173 (0.935-5.049) | 0.071 |
