# Supplementary material for: Carboxypeptidase E is a prognostic biomarker co-expressed with osteoblastic genes in osteosarcoma
Source: PeerJ. 2023 Aug 30;11:e15814. doi: 10.7717/peerj.15814 (PMC10474831; doi:10.7717/peerj.15814)
Supplement: Supplemental Information 6 [file peerj-11-15814-s006.docx]

Table S3 Univariate analysis and Multivariate analysis of relapse-free survival

| Characteristics | Total(N) | Univariate analysis | |  | Multivariate analysis | |
| --- | --- | --- | --- | --- | --- | --- |
|  |  | Hazard ratio (95% CI) | P value |  | Hazard ratio (95% CI) | P value |
| Metastasis | 99 |  |  |  |  |  |
| No | 75 | Reference |  |  |  |  |
| Yes | 24 | 2.625 (1.491-4.622) | **<0.001** |  | 1.776 (0.819-3.852) | 0.146 |
| Tumor region | 63 |  |  |  |  |  |
| Distal | 36 | Reference |  |  |  |  |
| Other&Proximal&Proximal & Distal | 27 | 0.429 (0.204-0.903) | **0.026** |  | 0.528 (0.244-1.143) | 0.105 |
| Age | 99 |  |  |  |  |  |
| <18 | 76 | Reference |  |  |  |  |
| >=18 | 23 | 0.464 (0.209-1.028) | 0.058 |  | 0.394 (0.150-1.040) | 0.060 |
