# Supplementary material for: Carboxypeptidase E is a prognostic biomarker co-expressed with osteoblastic genes in osteosarcoma
Source: PeerJ. 2023 Aug 30;11:e15814. doi: 10.7717/peerj.15814 (PMC10474831; doi:10.7717/peerj.15814)
Supplement: Supplemental Information 7 [file peerj-11-15814-s007.docx]

**Miame Checklist**

Part 1 Experiment description

1. Mouse type: Not covered
2. experimental variables :osteosarcoma tissues vs. adjacent normal tissues
3. n-count: 12
4. other variables: Not covered

Part 2 Array design.

1. Total RNA was quantified by NanoDrop ND-2000 (Thermo Scientific) and RNA integrity was analyzed using Agilent Bioanalyzer 2100 (Agilent Technologies).
2. STRING (https://string-db.org/) was employed to predict interactions between functional proteins and to establish a PPI network. The network was conducted with Cytoscape software (version 3.9.1). The PPIs between the putative protein biomarkers were analyzed using the STRING web service database (https://string-db.org/).

Part 3 Samples

1. Cy3/Cy5 labels for tissues: Not covered
2. Dye swap? Or reference control?：not covered

3. the source of the original sample:osteosarcoma tissues and adjacent normal tissues collected from the Department of Orthopaedic Oncology Surgery of the Beijing JiShuiTan Hospital.

4.the technical extraction of the nucleic acids：TRIzol reagent (Invitrogen) was using to extract RNA based on the manufacturer’s protocol (Invitrogen).

Part 4 Hybridizations

Not covered

Part 5 Measurements

1. The relative mRNA levels were normalized against the housekeeping gene GAPDH (NM_001357943.2).

Part 6 Normalization controls
